# Electroencephalographic findings among inpatients with COVID-19 in a tertiary hospital from a middle-income country

**DOI:** 10.1590/0004-282X-ANP-2020-0555

**Published:** 2021-05-08

**Authors:** Luíza Alves CORAZZA, João Fellipe Santos TATSCH, Maraysa Pereira BARROS, Apolônio Peixoto de QUEIROZ, Luana Lôbo Ribeiro BATISTA, Mariana Barbosa AIDAR, Meire Argentoni BALDOCCHI, Maria Sheila Guimarães ROCHA, Sonia Maria Dozzi BRUCKI

**Affiliations:** 1 Hospital Santa Marcelina, Departamento de Neurologia, São Paulo SP, Brazil. Hospital Santa Marcelina Departamento de Neurologia São Paulo SP Brazil

**Keywords:** Electroencephalography, Betacoronavirus, Encephalopathy, Status Epilepticus, Eletroencefalografia, Betacoronavírus, Encefalopatias, Estado Epiléptico

## Abstract

**Background:**

In 2019, the world witnessed the emergence of a new type of coronavirus - the severe acute respiratory syndrome coronavirus 2 (SARS-CoV-2). The spectrum of coronavirus disease 2019 (COVID-19) is variable, and amongst its manifestations are neurological implications.

**Objective:**

This report aimed to describe electroencephalographic findings in COVID-19 patients from a general tertiary hospital in São Paulo, Brazil.

**Methods:**

It was a retrospective, observational, and non-interventional study. Data were collected anonymously, comprising inpatients from Mar 1 to Jun 30, 2020, either confirmed (positive RT-PCR) or probable cases (CO-RADS 4/5) who had performed EEG during hospitalization.

**Results:**

Twenty-eight patients were enrolled, 17 (60.7%) women and 11 men, with a median age of 58 (minimum and maximum: 18-86; IQR 23.5). COVID-19 diagnosis was confirmed in 22 (78.5%). Twenty-one patients (75%) had severe disease, requiring mechanical ventilation due to acute respiratory distress syndrome (ARDS); 16 (57.1%) patients developed adjunct sepsis throughout hospitalization. There was no specific pattern found for COVID-19 in EEG. No patients presented with *status epilepticus* or electrographic events; most patients developed an encephalopathic pattern, as seen in most studies, with a high prevalence of altered mental status as an indication for EEG. Adjunct sepsis was associated with higher mortality.

**Conclusions:**

EEG presents as a useful tool in the context of COVID-19, as in other conditions, to differentiate nonconvulsive status epilepticus (NCSE) from encephalopathy and other causes of mental status alterations. Further studies are required to analyze whether there might be a specific EEG pattern to the disease.

## RESUMO

**Antecedentes:**

Em 2019, testemunhou-se o surgimento de um novo tipo de coronavírus - o coronavírus associado à síndrome da angústia respiratória tipo 2 (SARS-CoV-2). O espectro da doença associada ao novo coronavírus, a COVID-19, é variável e, dentre suas manifestações, há implicações neurológicas.

**Objetivos:**

O presente estudo objetivou descrever achados eletroencefalográficos em pacientes com COVID-19 internados em um hospital terciário em São Paulo.

**Métodos:**

Tratou-se de estudo observacional, retrospectivo e não-intervencionista, realizado por meio de coleta anônima e retrospectiva de dados de prontuário médico de pacientes com diagnóstico confirmado (RT-PCR positivo) ou provável (CO-RADS 4 ou 5), que realizaram eletroencefalograma durante internação hospitalar.

**Resultados:**

Vinte e oito pacientes foram elencados, 17 (60,7%) mulheres e 11 homens. O diagnóstico de COVID-19 foi confirmado em 22 (78,5%) dos casos. Dos pacientes, 21 (75%) apresentaram a doença, requerendo suporte ventilatório, e 16 (57,1%) desenvolveram sepse sobreposta. Não houve padrão específico de EEG para COVID-19, e nenhum paciente apresentou estado de mal epiléptico ou crise eletrográfica; a maioria desenvolveu padrão de encefalopatia, com alentecimento da atividade cerebral, sendo a alta prevalência de alteração de estado mental a indicação para o exame. A sepse sobreposta foi associada a um pior desfecho, com maior mortalidade.

**Conclusão:**

No contexto da COVID-19, o EEG figura como ferramenta importante, auxiliando, como em outras condições, na diferenciação entre estado de mal epiléptico, encefalopatia e outras causas de alteração do estado mental. Estudos adicionais são necessários para avaliar a existência de padrão específico de alteração eletroencefalográfica na COVID-19.

## INTRODUCTION

In 2019, the world witnessed the emergence of a new type of coronavirus - the severe acute respiratory syndrome coronavirus 2 (SARS-CoV 2) -, which rapidly spread, giving rise to a pandemic. The spectrum of coronavirus disease 2019 (COVID-19) is extremely variable, ranging from asymptomatic individuals to severe acute respiratory distress^1^. Some COVID-19 neurological implications are acute cerebrovascular disease, encephalitis and encephalomyelitis, encephalopathy, seizures, peripheral nervous system, muscle diseases, headache, and dizziness^2^. In this context, electroencephalogram (EEG) figures as a useful tool to differentiate encephalopathy from nonconvulsive epilepticus status.

This paper aimed to describe electroencephalographic findings in COVID-19 patients from a general tertiary hospital in São Paulo, Brazil.

## METHODS

It was a unicentric, retrospective, observational, and non-interventional study approved by the hospital’s ethical committee, under CAAE: 37098820.3.0000.0066, following the Declaration of Helsinki and as part of a project to investigate neurological manifestations of COVID-19^3^.

Data were collected anonymously from medical records of inpatients from Mar 1^st^ to Jun 30^th^, 2020, who were either COVID-19 confirmed cases - through positive reverse transcription polymerase chain reaction (RT-PCR) - or highly probable cases, which were those with negative RT-PCR but compatible clinical features and computerized thoracic tomography (CT) - CO-RADS 4 or 5^4^, which performed EEG during hospitalization. Our center did not allow additional RT-PCR testing in individuals with a previous negative test with a compatible CT scan.

Analyzed data comprised demographic characteristics, comorbidities, mechanical ventilation, sedation, use of antiepileptic drugs during EEG, and EEG indication and findings.

Routine EEG was performed using scalp electrodes, placed according to the International 10-20 System, and filters were set with high-pass at 0.5 Hz and low-pass at 70 Hz.

Two clinical electroencephalographers, who had access to clinical data consisting of sedation, use of antiepileptic drugs, and description of abnormal movements during the exam, if present, reviewed EEGs.

Statistical analysis was performed using the Action Stat software. Proportions, median values, and Interquartile Range (IQR) were calculated for descriptive analysis. Data were compared using Fisher’s exact test with a significance level of p<0.05.

## RESULTS

Twenty-nine patients were initially registered, but one was excluded given the late result of negative PCR for COVID -19 and CO-RADS^4^ classification of less than 4.

Twenty-eight patients were enrolled, 17 (60.7%) women and 11 men, with a median age of 58 years (minimum and maximum: 18-86; IQR 23.5). COVID-19 diagnosis was confirmed in 22 (78.5%) of them. Twenty-one patients (75%) had severe disease, requiring mechanical ventilation due to acute respiratory distress syndrome (ARDS), 17 (60.7%) acute kidney injury, and, of those, 13 needed hemodialysis. 16 (57.1%) patients developed adjunct sepsis throughout hospitalization. Three (10.7%) suffered cardiorespiratory arrest, and two (7.1%) had severe hypoxemia. Sixty-eight percent (n=19) had altered mental status, 25% (n=7) had both altered mental status and seizures, and 7.1% (n=8) had isolated seizures as clinical indication for EEG. Of those presented with clinical events, generalized tonic-clonic seizures occurred in seven patients (25%), a focal seizure happened in one (3.6%) patient, and generalized myoclonus ensued in one patient. Only two (7.1%) had epilepsy.

During EEG, 20 (71.4%) were not under sedation or antiepileptic drugs (AED), 6 (21.4%) were sedated (1 with fentanyl and ketamine, 1 with propofol and fentanyl, 1 with ketamine, fentanyl and midazolam, 2 with midazolam and fentanyl, and 1 with midazolam), and 8 (28.6%) were under AED - 5 (17.9%) in monotherapy (1 with phenytoin, 2 with midazolam, 1 with propofol and 1 with ketamine), 2 (7.1%) used 2 drugs (1 used ketamine and midazolam, 1 used phenytoin and midazolam), and 1 (3.6%) used 3 drugs - phenytoin, phenobarbital, and clobazam.

**Figure 1.**
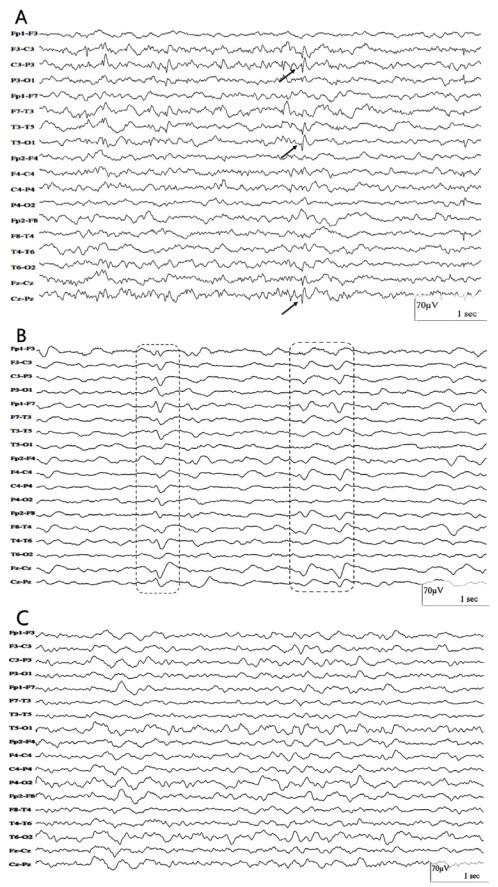
(A) Woman, 21 years old. Electroencephalogram shows sharp waves over the left hemisphere and midline (arrows). (B) Man, 57 years old. Electroencephalogram shows slow waves with triphasic morphology (highlighted in dashed boxes). (C) Woman, 38 years old. Electroencephalogram shows moderately disorganized background activity with bursts of irregular delta waves.

Regarding EEG findings concerning background activity, results are described in Table 1. One subject, who had epilepsy, showed posterior bilateral epileptiform discharges, predominating on the left side (Figure 1). None of the patients had electrographic seizures or *status epilepticus*.

**Table 1.** Electroencephalogram results - background alterations.

EEG	Frequency	Percentage
Normal	5	17.9
Predominant theta activity	10	35.7
Burst-suppression	1	3.6
Slow background posterior activity <8 Hz	3	10.7
Triphasic waves	2	7,1
Diffuse attenuation	7	25.0
Total	28	100.0

EEG: electroencephalogram.

Sixteen (57.1%) participants died during the study, and 12 (42.9%) were discharged, with a median time of hospitalization of 21 days (minimum 6, maximum 67; IQR 27.8).

**Figure 2.**
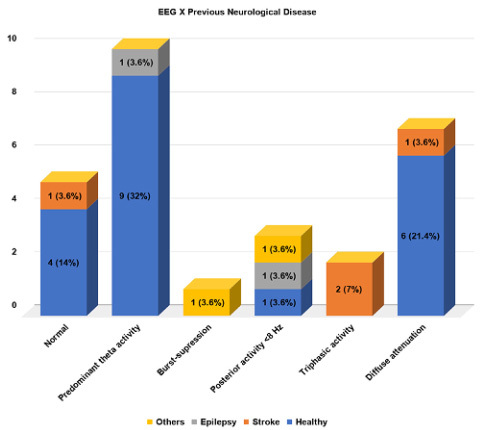
Electroencephalographic findings and their relationship with previous neurological disease in the population.

**Figure 3.**
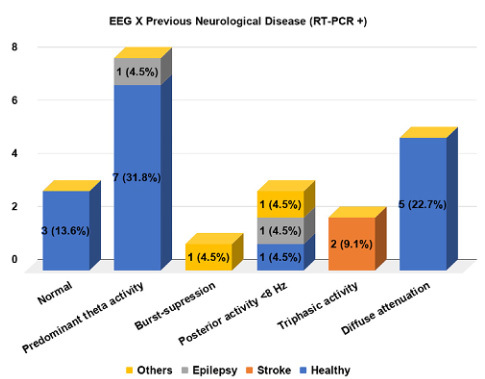
Electroencephalographic findings and their relationship with previous neurological disease in RT-PCR positive patients.

There was an association with the presence of a previous neurological diagnosis and EEG results (Figure 2), in which a higher prevalence of predominant theta activity (90%) and diffuse attenuation (85%) were found in patients with no previous disease. Triphasic morphology (Figure 1) was found only in patients with previous stroke (one with Wallenberg’s syndrome and the other with multiple subcortical internal border zone small infarctions on computerized tomography). Normal EEG was also more prevalent in patients with no previous neurological diagnosis (80%). These associations were also true when a sub analysis of positive COVID-19 patients was made (Figure 3).

There was no association between EEG results and clinical complications: sepsis (p=0.22), acute kidney injury (p=0.38), hemodialysis (p=0.33), and cardiac arrest (p=0.51), as well as the use of sedation (p=0.18) and AED (p=0.11). No relation was observed between EEG and positive RT-PCR (p=0.89).

A sub analysis concerning these same variables and only patients with confirmed diagnosis through RT-PCR was performed. A similar result was found, with no statistical significance for the same analysis: sepsis (p=0.65), acute kidney injury (p=0.85), hemodialysis (p=0.64), cardiac arrest (p=0.71), sedation (p=0.36), and AED (p=0.25).

Patients’ comorbidities (systemic arterial hypertension, diabetes mellitus, dyslipidemia, cancer, immunosuppression, smoking habits, previous neurological disease, and final neurological diagnosis) were also cross-tabulated with EEG results. There was statistical significance (p<0.05) concerning previous neurological disorders, both when all patients were considered (p=0.004) and when only COVID-19 positive RT-PCR patients were sub analyzed (p=0.003).

Amongst patients with COVID-19 positive RT-PCR, there was a higher prevalence of encephalopathy as the final neurological diagnosis - 13 (59%) *versus* no patients in the RT-PCR negative group. In contrast, in those with negative RT-PCR but with compatible clinical features and CT scan, there was a higher prevalence of ischemic stroke (50 *versus* 18%), hemorrhagic stroke (33 *versus* 0%), and acute symptomatic seizure (17 *versus* 9%).

## DISCUSSION

COVID-19 is a multifaceted disease, ranging from asymptomatic individuals to ARDS^5^. Multiple neurological implications have been reported to date, initially by Mao et al.^6^, who described acute cerebrovascular disease, impaired consciousness, muscle disease, and peripheral nervous system disease. Later, cases of encephalitis and encephalomyelitis^7^, encephalopathy^8^, seizures, headache, dizziness, and psychosis were additionally reported^9^.

Neurological complications of COVID-19 may be caused by many concomitant factors: endothelial lesion, prothrombotic state, inflammatory storm, and sequelae of systemic complications^10^^,^^11^^,^^12^.

 In the specific context of the direct viral action on the central nervous system, resulting in neurological symptoms, even though the majority of the analyzed cases did not present with RT-PCR positivity in cerebrospinal fluid^7^^,^^10^^,^^11^^,^^12^^,^^13^^,^^14^^,^^15^^,^^16^ (either because of no availability of testing at the time, or low sensitivity - in one case, when repeated, results came out positive^17^), two main pathways might be theorized: the targeting of the angiotensin-converting-enzyme-2 receptors, which are heavily present in the central nervous system, including brain cells, glial cells, and endothelial cells of the blood-brain barrier; or through the olfactory nerve, causing inflammation and demyelination^1^^,^^18^^,^^19^.

It has come to notice that many patients with COVID-19 in intensive care units have presented delayed awakening and altered mental status, which may be multifactorial due to metabolic disorders, renal failure, hypoxemia, adjunct sepsis, encephalitis, cerebrovascular events, severe encephalopathy, and nonconvulsive status epilepticus^11^^,^^16^.

In this study, we examined multiple complications of the disease, and their incidence was independent of EEG results, which was also valid for the patients with positive COVID-19 RT-PCR. That could be related to the fact that no specific electroencephalographic pattern was found to the disease in our population.

Compared to the literature, the analyzed population presented a higher incidence of mental status alterations as an indication for the exam: 93 *versus* 35^20^, 65^21^, 77.3^22^, 90^23^, and 61.7%^24^. EEG is usually ordered for patients with this clinical condition in our center, although this has also been a frequent indication in other studies.

This population also presented with a decreased occurrence of epileptic discharges in EEG compared to previous publications - 1 subject (3,6 *versus* 21^25^, 19^21^, 40.9^16^, and 11%)^26^.

Concerning EEG findings, compared to a recent review^24^, which analyzed 84 studies - totaling a population of 617 subjects - our patients presented with similar alterations considering background activity. The prevailing finding was diffuse slowing (10; 35.7% *versus* 423; 68.6%). A higher percentage of slow posterior background activity (3; 10.7% *versus* 13; 2.1%), attenuation (7; 25% *versus* 8; 1.3%), as well as burst-suppression pattern (1; 3.6% *versus* 13; 2.1%) were found, which are related to the size of the sample. Regarding periodic and rhythmic patterns, triphasic morphology was observed in 2 (7.1%) *versus* 18 patients (2.9%) in the review; no other periodic patterns were observed in this population. Thirteen patients (46.4%) in our sample were diagnosed with encephalopathy, which is compatible with the EEG findings in this population and in most studies.

One patient, who was previously diagnosed with epilepsy, presented posterior bilateral epileptiform discharges, predominating on the left side (3.6%), *versus* 35 focal epileptiform discharges in the review^24^ (5.7%). It is impossible to blame COVID-19 exclusively for this alteration, but it is reasonable to assume that the illness could contribute to this finding.

No patient in the sample presented with *status epilepticus* or frontal epileptiform discharges, which have been proposed as biomarkers for COVID-19^24^, given their apparent predominance in focal discharges^1^^,^^8^^,^^12^^,^^16^^,^^20^^,^^26^^,^^27^^,^^28^^,^^29^^,^^30^^,^^31^^,^^32^^,^^33^ and originating *status epilepticus*.

Whereas it was not possible to define a specific EEG pattern to the encephalopathy related to COVID-19^11^^,^^34^ using routine EEG, Pastor et al.^23^, through quantitative EEG, found in their population that the raw EEGs showed a nearly physiological pattern. The mean spectra display the existence of a significant encephalopathic pattern with an excess of generalized delta activity and lower alpha and beta values. The distribution of bands demonstrated higher relative amounts of faster bands (α and β). Synchronization was different for COVID patients’ EEGs when compared to other toxic encephalopathies and post-cardiac arrest.

EEG monitoring in the context of COVID-19 may be crucial to identify, for instance, focal lesions decurrent of hypoxemia, focal epilepsies^35^ or focal status epilepticus as a primary manifestation of the disease^36^, or even a new-onset status epilepticus^31^ and frontal encephalopathy^27^, as well as alpha coma patterns^10^^,^^11^.

To date, there has been no robust evidence to associate EEG results with prognostic factors, even though Skorin et al.^37^ found in their population that the presence of cancer and the need for an electroencephalographic study during the third week of COVID-19 evolution were independent risk factors for mortality. In our sample, adjunct sepsis led to a more unsatisfactory outcome among the various complications of the disease.

There were limitations to this study, considering its retrospective design, patients’ critical statuses - therefore the high mortality in the analyzed population - as well as there was no definite protocol for EEG ordering in all COVID-19 inpatients in our hospital. Thus, only those who had undergone the exam were analyzed, henceforth the small sample.

Nonetheless, an important role for EEG in COVID-19 patients was observed, for the diagnosis of encephalopathy and differentiation from *status epilepticus* and other causes of mental status alterations, as in other diseases, and to better understand the central nervous system implications of this new virus, as well as, perchance, define a specific EEG pattern, both qualitative and quantitively, with a larger population and further analysis.
